# Supplementary material for: Generation of Multipotent Stem Cells from Adult Human Peripheral Blood Following the Treatment with Platelet-Derived Mitochondria
Source: Cells. 2020 May 29;9(6):1350. doi: 10.3390/cells9061350 (PMC7349571; doi:10.3390/cells9061350)
Supplement: Supplementary file 1 [file cells-09-01350-s001.pdf]

# Figure S1

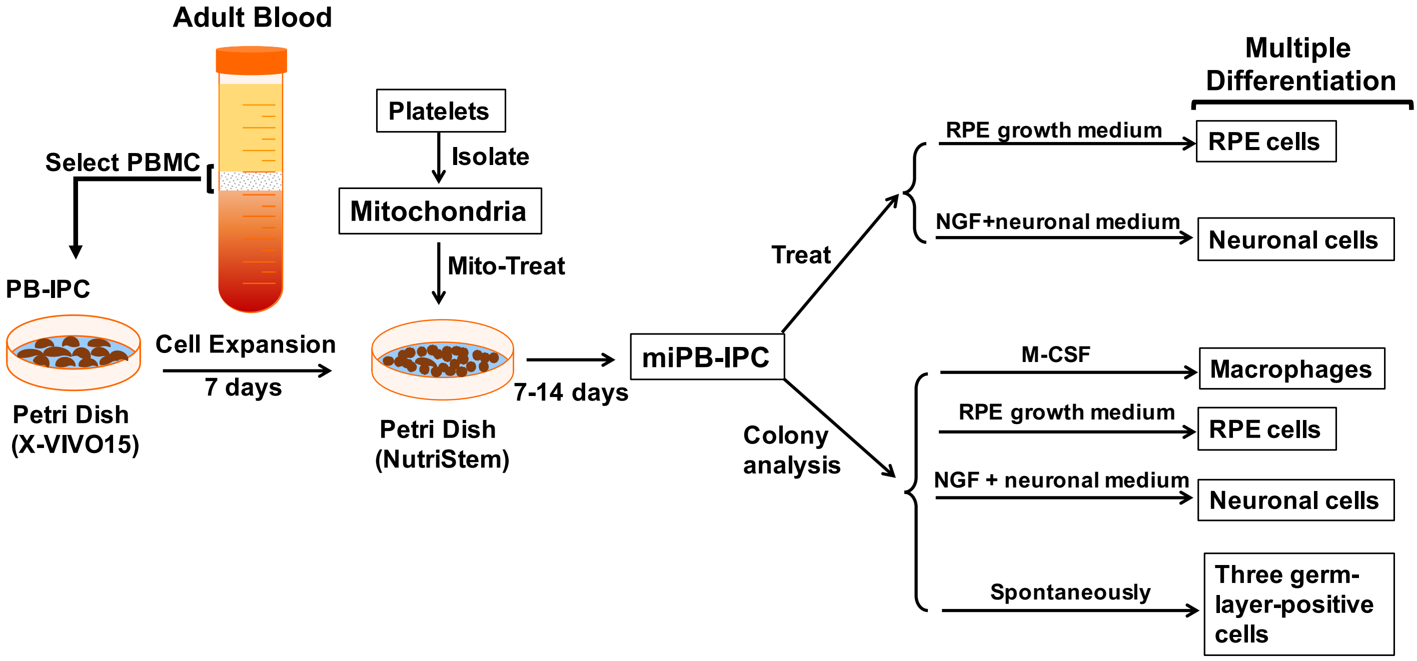


**Figure S1. Outline of the whole protocol from the generation of miPB-IPC to multipotent cellular differentiation of miPB-IPC.** Briefly, mononuclear cells (PBMC) were isolated from buffy coats blood using Ficoll-Paque™ PLUS (g=1.007), followed by removing the red blood cells using Red Blood Cell Lysis buffer. After three washes with saline, the whole PBMC were seeded in 150 × 15mm Petri dishes (BD Falcon) at 1 × $10^6$ cells/ml, 25ml/dish in chemical-defined serum-free culture X-VIVO 15™ medium (Lonza) without adding any other growth factors and incubated at 37 °C in 8% $CO_2$. Seven days later, PB-IPC were growing and expanded by adhering to the hydrophobic bottom of Petri dishes. Consequently, PB-IPC were washed three times with saline and all floating cells were removed, and followed by adding the serum-free NutriStem® hPSC XF culture medium (Corning) for continue cell culture in the presence of 100 μg/ml mitochondria, at 37 °C in 8% $CO_2$. The mito-treated PB-IPC (miPB-IPC) were usually applied for experiments during 7-14 days.

# Figure S2

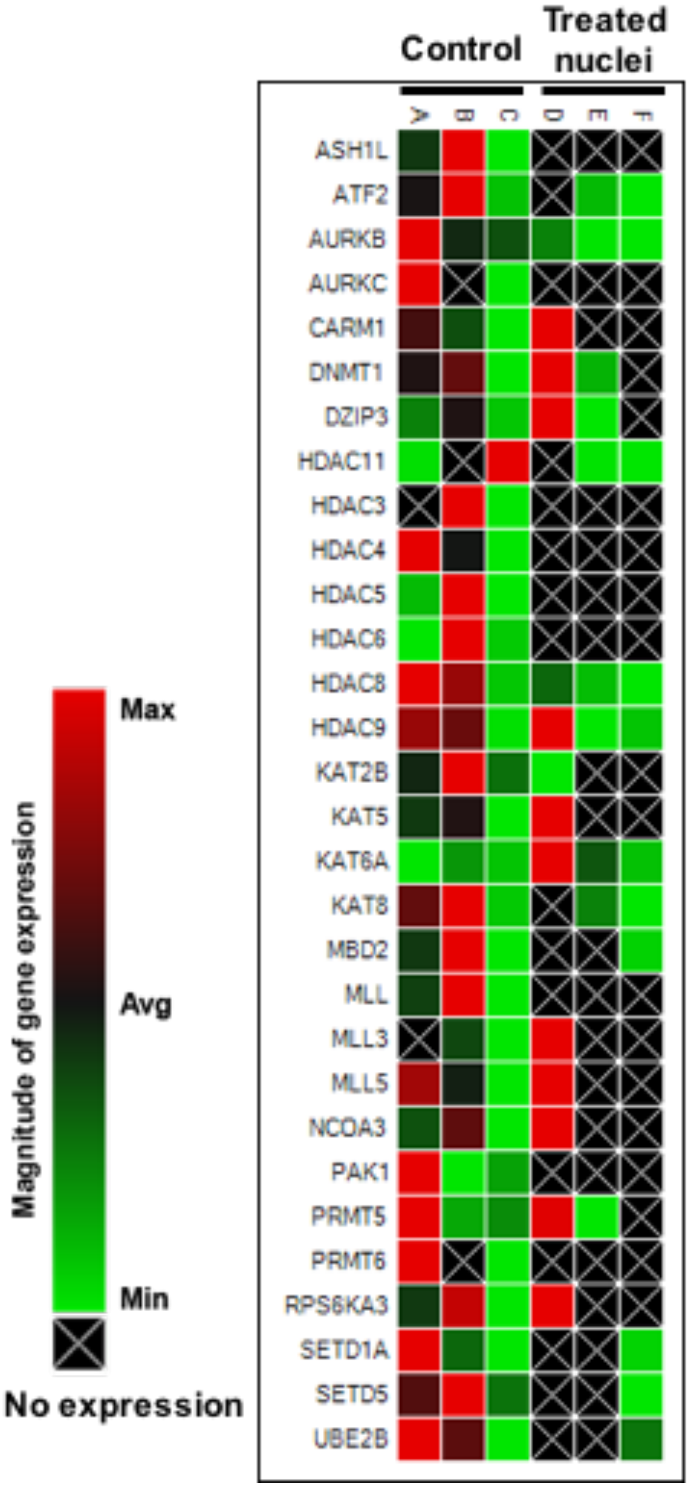


**Figure S2 Real time PCR array Epigenetic Chromatin Modification Enzyme**
The purified nuclei from PB-SC show markedly epigenetic changes post treatment with exogenous Mitochndria for 4 hrs. To determine the direct modulation of mitochondria on nucleus, the purified PB-SC-derived nuclei were treated with isolated mitochondria for 4 hrs under $37^0$C, 5% $CO_2$ condition, and followed by real time PCR array. The data uncovered the marked changes in epigenetic chromatin modification enzyme-related genes including DNA methyltransferase 1 (DNMT1), histone acetyltransferases [activation transcription factor-2 (ATF2), lysine acetyltransferase 2B (KAT2B), KAT5, and KAT8], histone methyltransferases [coactivator-associated arginine methyltransferase 1 (CARM1), mixed lineage leukemia protein (MLL), MLL3, Protein arginine methyltransferase 5 (PRMT5), and PRMT6], histone methyltransferase activity-associated SET (Su (var), Enhancer of Zeste and Trithorax) domain proteins [ASH1L (absent, small, or homeotic)-like (Drosophila), SET domain containing 1A (SETD1A), and SETD5], histone phosphorylation [(aurora kinase B (AURKB), AURKC, p21 protein-activated kinase 1 (PAK1), and ribosomal protein S6 kinase polypeptide 3 (RPS6KA3)), Histone ubiquitination [DAZ interacting protein 3(DZIP3) and ubiquitin-conjugating enzyme E2B (UBE2B)], DNA and histone demethylase methyl-CpG binding domain protein 2 (MBD2), and histone deacetylases [histone deacetylase 3 (HDAC3), HDAC4, HDAC5, HDAC6, HDAC8, HDAC9, and HDAC11]].

# Figure S3

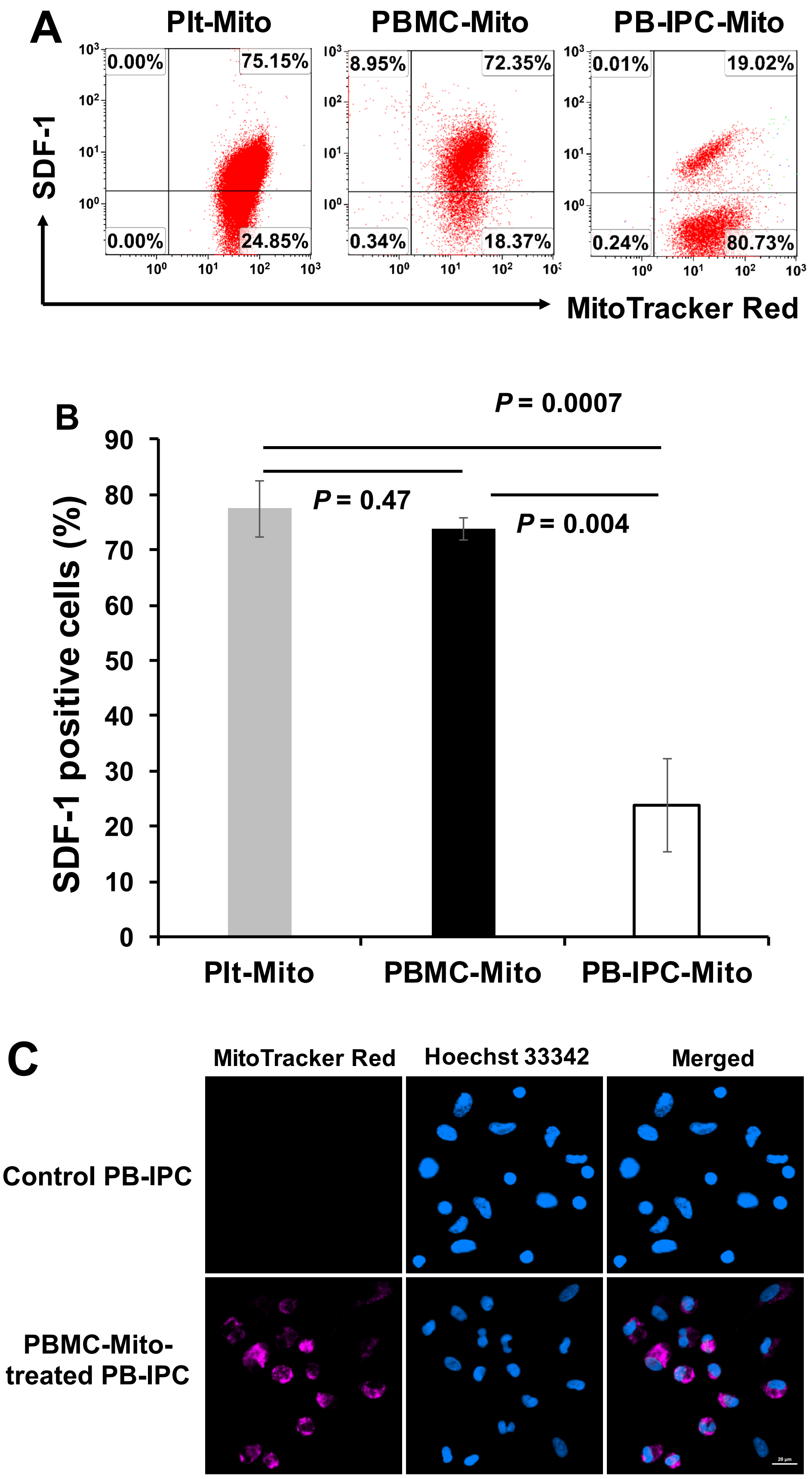


**Figure S3. Compare the SDF-1 expression among platelet-derived mitochondria and PBMC-derived mitochondria and PB-IPC-derived mitochondria.** The mitochondria were isolated from peripheral blood (PB)-platelets and PBMC and PB-IPC respectively using the Mitochondria Isolation kit (Thermo scientific) according to the manufacturer's recommended protocol. **(A)** Flow cytometry show the expression of SDF-1 on the platelet-derived mitochondria and PBMC-derived mitochondria and PB-IPC-derived mitochondria. Isotype-matched IgGs served as controls. Data were representative from three experiments. **(B)** Compare the level of SDF-1 expression among platelet-derived mitochondria (Plt-Mito) and PBMC-derived mitochondria (PBMC-Mito) with no marked difference, but much lower on the PB-IPC-derived mitochondria (PB-IPC-Mito). Data represent mean ± s.d., N = 3. **(C)** Confocal microcopy shows the penetration of PBMC-derived mitochondria into the nucleus of PB-IPC. PB-IPC were treated with MitoTracker Red-labeled PBMC-derived mitochondria (100 µg/ml) at 37 °C in 5% $CO_2$. After the treatment for 4 – 6 hours, cells were observed and photographed with Nikon A1R confocal microscope on Nikon Eclipse Ti2 inverted base, using software NIS Elements Version 4.60.
